# RNase Hybridization-Assisted Amplification (RHAM) Technology: A High-Sensitivity, Field-Deployable Alternative to Quantitative Polymerase Chain Reaction for the Rapid Detection of African Swine Fever Virus

**DOI:** 10.3390/vetsci12111068

**Published:** 2025-11-07

**Authors:** Sakchai Ruenphet, Nitipon Srionrod, Teera Nipakornpun, Supphathat Wutthiwitthayaphong

**Affiliations:** 1 Animal Biotechnology, Mahanakorn University of Technology, 140 Cheum-Sampan Rd., Nong Chock, Bangkok 10530, Thailand; sakchai@mut.ac.th; 2 Immunology and Virology Department, Mahanakorn University of Technology, 140 Cheum-Sampan Rd., Nong Chock, Bangkok 10530, Thailand; 3 Clinic for Swine, Mahanakorn University of Technology, 140 Cheum-Sampan Rd., Nong Chock, Bangkok 10530, Thailand; nitipon@mut.ac.th; 4 Pacific Biotech, 42 Moo 4, Petchaboon-Chalianglub Rd., Napa, Muang, Petchaboon 67000, Thailand; teera@brianet.com

**Keywords:** African swine fever virus, immunochromatographic assay, quantitative polymerase chain reaction, RNase hybridization-assisted amplification

## Abstract

African Swine Fever (ASF) control is severely limited by a diagnostic gap, as laboratory-based quantitative polymerase chain reaction (qPCR) is highly sensitive but slow, whereas field-deployable immunochromatographic assays (ICAs) are rapid but unreliable. To address this limitation, this study evaluated a novel, rapid isothermal assay, RNase hybridization-assisted amplification (RHAM), as a high-sensitivity, point-of-need diagnostic solution. This study compared the performance of RHAM and a conventional p72-based ICA against the qPCR reference standard using 106 diverse clinical field samples, including oral swabs, blood, serum, and organs, collected from suspected ASF cases in Thailand. The ICA exhibited markedly low diagnostic performance, achieving only 56.76% sensitivity and showing moderate agreement (κ = 0.421) with qPCR, highlighting the need for a more reliable alternative. In contrast, the RHAM assay achieved 94.59% sensitivity and 96.88% specificity, providing results rapidly within 35 min. This statistically superior performance (McNemar’s test, *p* < 0.0001) demonstrated almost perfect agreement (κ = 0.891) with the qPCR reference standard, missing only four samples with very high Ct values (>30). In conclusion, RHAM is a powerful, accurate, and field-deployable diagnostic tool that effectively bridges the diagnostic gap, offering qPCR-like sensitivity for the rapid containment of ASF outbreaks.

## Simple Summary

African swine fever is a devastating and costly pig disease with no vaccine, making fast and accurate testing essential to stop its spread. Current rapid on-farm tests are often unreliable and miss infected animals, while accurate lab tests are too slow for effective outbreak control. This study aimed to evaluate a new, portable testing technology, RNase hybridization-assisted amplification, to determine if it could provide a better solution for on-site diagnosis. We compared its performance against a common test strip and the best available laboratory method using real samples from pig farms in Thailand. Our results showed the new test was highly effective, correctly identifying nearly 95% of infected pigs, which is almost as accurate as the laboratory test, while delivering results in under 35 min. In contrast, the standard test strip missed over 40% of infected pigs. We conclude that this new technology is a powerful and reliable tool for on-farm diagnosis. Its adoption will be valuable to society by empowering veterinarians and authorities to quickly detect the disease, prevent its spread, and protect the livelihoods of farmers and the global pork supply.

## 1. Introduction

African swine fever (ASF) is a devastating hemorrhagic disease capable of causing mortality rates approaching 100% in domestic pigs, posing a catastrophic threat to the global swine industry [1,2]. Since its transcontinental expansion began accelerating in 2018, the virus has rapidly spread across Asia, Europe, and. The Americans, resulting in the loss of hundreds of millions of pigs and causing severe economic destabilization, disproportionately affecting small-scale farmers and threatening global food security [3,4,5,6]. The disease is caused by the African swine fever virus (ASFV), and its complex epidemiology, combined with the absence of a commercially available effective vaccine, makes control exceptionally challenging [3,4,5]. Consequently, management strategies rely heavily on strict biosecurity, culling of infected herds, and robust surveillance to prevent further spread [7]. Recognizing its global significance, the World Organization for Animal Health (WOAH) lists ASF as a notifiable disease [8].

Effective control of ASF is contingent on surveillance programs that ensure early, rapid, and accurate detection of the virus [9]. According to international guidelines from WOAH and Food and Agriculture Organization (FAO), a definitive. ASF diagnosis must be based on laboratory confirmation [10]. Quantitative polymerase chain reaction (qPCR) is the designated gold-standard method of choice, valued for its high sensitivity and specificity [8]. However, qPCR requires specialized laboratory equipment and highly trained personnel, often resulting in substantial logistical delays between sampling on the farm and receiving a diagnosis [11]. This logistical bottleneck frequently hampers the timely containment efforts necessary to control an outbreak.

To address the need for faster, on-site results, rapid field-deployable tests have been developed. Among the most common are immunochromatographic assays (ICAs), or lateral flow devices, which detect viral proteins like p72 [12]. While their simplicity, portability, and speed make them useful for preliminary screening during active outbreaks in resource-limited areas, international guidelines mandate that all field-based results, particularly negatives, must be confirmed by laboratory testing (i.e., qPCR) [9]. This is because ICAs notoriously suffer from limited diagnostic sensitivity, increasing the risk of false negatives, especially in animals with low viral loads during early infection or in asymptomatic carriers [10].

These contrasting limitations—the slow turnaround of highly accurate lab tests versus the low reliability of rapid field tests—highlight an urgent need for a diagnostic tool that bridges this gap, combining the high analytical sensitivity of qPCR with the speed and portability of an ICA. Isothermal amplification technologies, such as loop-mediated isothermal amplification (LAMP), have emerged as promising alternatives, as they enable rapid nucleic acid amplification without the need for complex thermal cycling equipment [13]. However, many traditional LAMP assays still require separate nucleic acid extraction steps or nuanced result interpretation. The novel RNase hybridization-assisted amplification (RHAM) technology evaluated in this study builds upon the isothermal principle but is designed to improve field usability by integrating sample lysis, amplification, and a simple fluorescent readout into a single, portable ‘sample-to-answer’ device. Therefore, this study evaluates the performance of this novel RHAM assay and a conventional ICA, comparing their diagnostic accuracy against the qPCR reference standard using a diverse set of clinical field samples from commercial farms in Thailand.

## 2. Materials and Methods

### 2.1. Ethical Approval

The animal procedures were approved by the Animal Research Ethics Committee of the Faculty of Veterinary Medicine, Mahanakorn University of Technology, Thailand (Approval code: ACUC-MUT-2024/004). All samples used in this study were obtained from field outbreaks or diagnostic submissions.

### 2.2. Sample and Sample Preparation

A total of 106 samples were collected from pigs with suspected or confirmed ASF to support diagnostic testing. The samples—comprising oral swabs, whole blood, serum, and internal organs—were obtained from commercial farms located in central and northeastern Thailand, including Nakorn Pathom, Ratchaburi, and Nakhon Ratchasima provinces. Sample selection was based on the presence of clinical signs consistent with ASF. All laboratory work was performed at the Virology and Molecular Diagnostic Center, Faculty of Veterinary Medicine, Mahanakorn University of Technology, Thailand.

For organ samples were collected from dead pigs. Pooled internal organs (lung, spleen, lymph nodes) were homogenized in phosphate-buffered saline (PBS), centrifuged at 2000× *g* for 10 min, and the supernatant was stored at −80 °C until analysis. For oral sampling, because a large sample volume was required for multiple testing methods, five swabs per pig were pooled into a conical tube containing 3 mL of PBS, vortexed, centrifuged at 2000× *g* for 10 min, and the resulting supernatant was stored at −80 °C until analysis. Blood samples were processed to obtain both whole blood and serum, which were stored at −80 °C until analysis.

### 2.3. Immunochromatography Assay

Monoclonal antibodies (mAbs) targeting the ASFV p72 protein were generated by Pacific Biotech Co., Ltd. (VetDiag^®^, Muang, Petchaboon, Thailand) under a patented process. Briefly, BALB/c mice were immunized intraperitoneally with p72 protein emulsified in Freund’s complete adjuvant (Sigma-Aldrich, St. Louis, MO, USA). Two booster injections were administered at two-week intervals. One week after the final booster, blood was collected to measure antibody titers, and the mouse with the highest titer received a final injection of p72 protein without adjuvant. Three days later, the spleen was harvested, and splenocytes were fused with myeloma cells using 50% polyethylene glycol (Sigma-Aldrich, St. Louis, MO, USA) at a 5:1 ratio. Hybridoma cells were cultured in Hypoxanthine-Aminopterin-Thymidine (HAT) selective medium (10^5^ cells/mL) in 96-well plates at 37 °C with 5% CO_2_. After 10 days, culture supernatants were screened for anti-p72 antibodies by indirect enzyme-linked immunosorbent assay (ELISA). Positive clones were subjected to limiting-dilution cloning to establish stable monoclonal cell lines.

**Figure 1:**
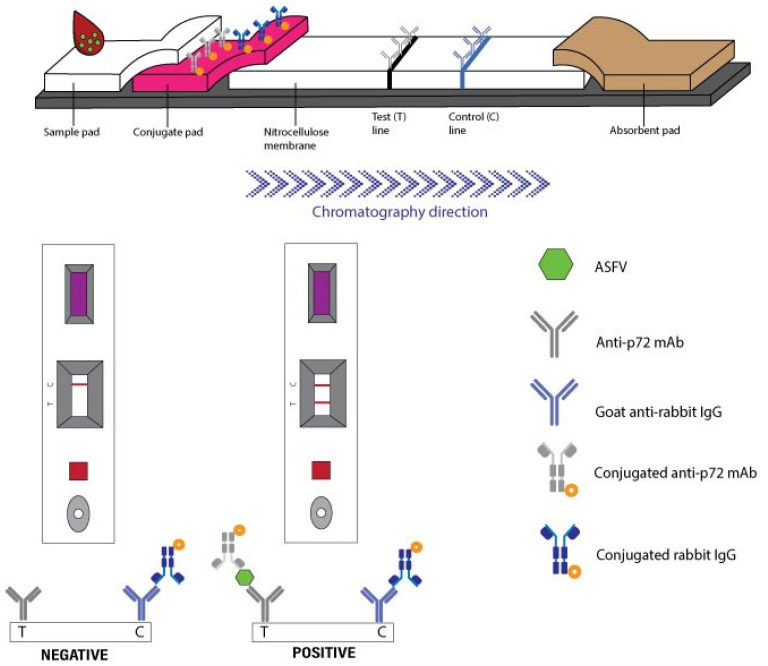
Schematic diagram of the p72 immunochromatography assay.

**Figure 2:**
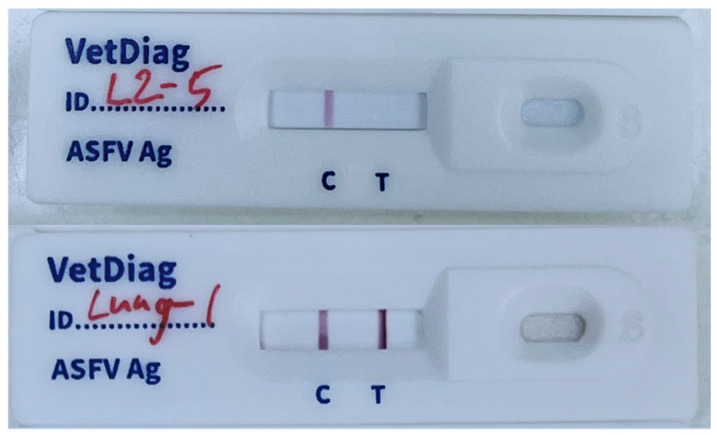
Exemplary immunochromatography assay results. Above shows a valid negative result, below shows a valid positive result.

The generated mAbs were subsequently used for ICA development. Nitrocellulose membranes were striped with goat anti-rabbit IgG (control line) and anti-p72 mAb (test line) at 1 μL/cm, then dried under <20% humidity for >4 h. Colloidal gold was prepared by boiling distilled water, adding 1% gold chloride trihydrate and 1% sodium citrate, and stirring until a red solution formed. The pH was adjusted to 7.2, and anti-p72 mAb was added dropwise and incubated for 30 min. The mixture was then blocked with 10% bovine serum albumin (BSA) and centrifuged at 8000× *g* for 30 min. The resulting pellet was resuspended in 1% BSA in PBS and stored at 4 °C. Conjugate pads were soaked in buffer containing sucrose, sodium azide, BSA, and gold-labeled antibodies (OD 2.0 for mAb, OD 0.7 for rabbit IgG), and then dried (>4 h, <20% humidity). Finally, the membranes, conjugated pads, and sample pads were assembled into plastic housings to produce the ICA kit (Figure 1 and Figure 2).

### 2.4. RNase Hybridization-Assisted Amplification (RHAM) Test

**Figure 3:**
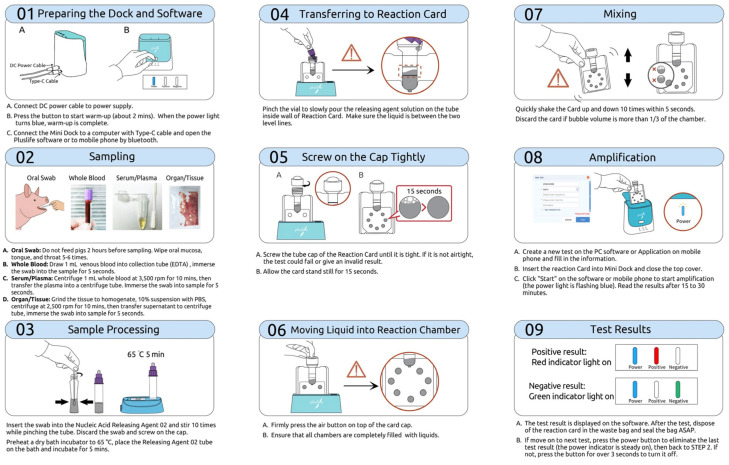
The operation process illustration of the RNase hybridization-assisted amplification (RHAM) test kit for nucleic acid detection of African swine fever virus.

**Figure 4:**
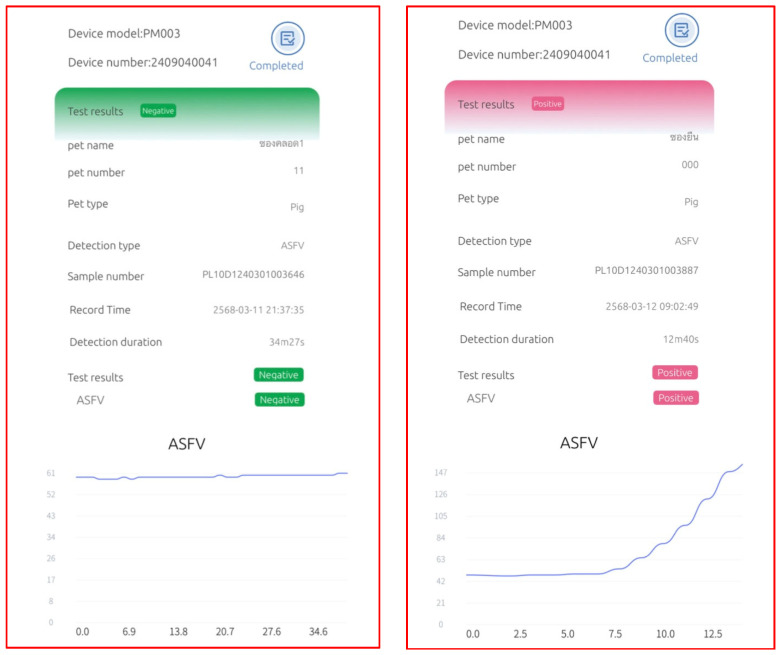
An exemplary RNase hybridization-assisted amplification (RHAM) test kit for nucleic acid detection of African swine fever virus (ASFV). The left panel shows a negative result, whereas the right panel shows a positive result. The results present fluorescence intensity curves recorded during the testing period, along with the corresponding detection duration. In ASFV-positive samples with high titers, the detection duration is shorter than in samples with low viral titers. In contrast, the detection duration for negative results is approximately 35 min.

The RHAM test kit comprises three main components: the Pluslife Integrated Nucleic Acid Testing Device, a nucleic acid releaser tube, and the Pluslife ASFV card (patented by Guangzhou Pluslife Biotech, Guangzhou, China). The procedure for ASFV detection is summarized as follows. Before testing, the device is preheated. A sample swab is then inserted into the nucleic acid releaser tube, twisted ten times while pinching the tip, and incubated at 65 °C for 5 min. The resulting lysate is poured into the Pluslife ASFV card between the injection lines. The card is placed on the Pluslife stand for 15 s, gently shaken, and then inserted into the device. Pressing the “Start Test” button initiate the amplification process. Test results are visually indicated: a red light denotes a positive ASFV result, whereas a blue light indicates a negative result. A schematic representation of the complete workflow is provided in Figure 3 and Figure 4 showed negative and positive results.

### 2.5. Quantitative Polymerase Chain Reaction

Viral DNA was extracted from all samples using the TAN Bead^®^ Nucleic Acid Extraction Kit (Taiwan Advanced Nanotech, Taoyuan, Taiwan) in conjunction with the Automated Nucleic Acid Extractor (Smart LabAssist SLA-E13200, Taiwan Advanced Nanotech). Quantitative detection of ASFV DNA was carried out using the Virotype^®^ ASFV 2.0 PCR Kit (Indical Bioscience, Leipzig, Germany) on a C1000 Touch Thermal Cycler (Bio-Rad, Hercules, CA, USA). The assay incorporates two internal controls: an endogenous β-actin control and an exogenous control added during DNA purification to verify both extraction efficiency and amplification integrity. The qPCR cycling conditions consisted of an initial denaturation at 95 °C for 2 min, followed by 40 cycles of 95 °C for 5 s and 60 °C for 30 s. Samples were interpreted as positive when the cycle threshold (Ct) value was <35, suspect when between 35 and 40, and negative when >40.

### 2.6. Statistical Analysis

Sensitivity, specificity, accuracy, and precision were evaluated by comparing the ICA and RHAM results with those obtained by qPCR, which served as the reference standard. Sample yielding results consistent with qPCR were categorized as true positives or true negatives, whereas discrepancies were classified as false positives or false negatives. The counts of each classification were then used to calculate diagnostic performance metrics according to standard evaluation methods previously described [14,15,16].

Sensitivity, specificity, accuracy, and precision for each diagnostic modality were calculated using the following formulas:

$$Sensitivity=\frac{No.\;of\;true\;positive}{No.\;of\;true\;positive+No.\;of\;false\;negative}\;\times \;100$$

$$Specificity=\frac{No.\;of\;true\;negative}{No.\;of\;true\;negative+No.\;of\;false\;positive}\times 100$$

$$Accuracy=\frac{\left(No.\;of\;true\;positive\right)+(No.\;of\;true\;negative)}{\left(No.\;of\;true\;positive\right)+\left(No.\;of\;true\;negative\right)+\left(No.\;of\;false\;positive\right)+(No.\;of\;false\;negative)}\times 100$$

$$Precision=\frac{No.\;of\;true\;positive}{No.\;of\;true\;positive+No.\;of\;false\;positive}\times 100$$

For all diagnostic methods evaluated, 95% confidence intervals (CIs) of sensitivity, specificity, accuracy, and precision were calculated using the Wilson score interval method for binomial proportions. This method was selected for its superior accuracy and reliability in estimating CIs, particularly for proportions derived from binary diagnostic outcomes (e.g., positive or negative results). To assess the agreement between each assay (ICA and RHAM) and the qPCR reference standard, Cohen’s kappa coefficient (κ) was calculated. Kappa values were interpreted according to the following thresholds: <0.20 (slight), 0.21–0.40 (fair), 0.41–0.60 (moderate), 0.61–0.80 (substantial), and 0.81–1.00 (almost perfect agreement). Furthermore, to directly compare the diagnostic performance—specifically sensitivity and specificity—of the ICA and RHAM assays using the same paired samples, McNemar’s test was employed. A *p*-value of <0.05 was considered statistically significant. All statistical analyses were conducted using GraphPad Prism version 9.0 (GraphPad Software, San Diego, CA, USA).

## 3. Results

A total of 106 field samples collected from different swine sources were tested for ASFV to evaluate the performance of an ICA and a novel RHAM assay against the reference qPCR method.

### 3.1. Distribution of Viral Load Across Sample Types

**Figure 5:**
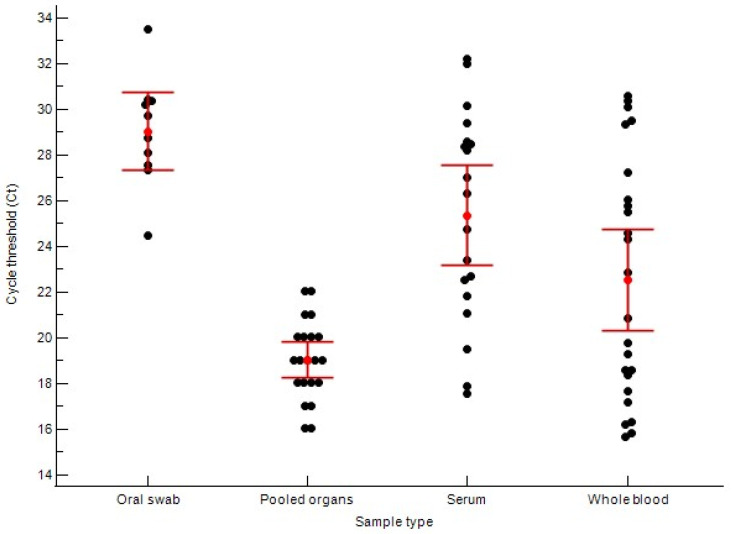
Scatter dot plot showing individual cycle threshold (Ct) values, means and standard deviation for qPCR-positive samples from oral swab (29.05 ± 2.41), pooled internal organs (19.04 ± 3.05), serum (25.37 ± 4.54) and whole blood (21.63 ± 5.24) (*n* = 74).

Among the 106 samples tested, 74 were positive for ASFV nucleic acid using the reference qPCR assay. Based on qPCR results, the viral load, inferred from the Ct values, varied significantly among different sample types. Tissue or organ samples exhibited the highest viral load, with a mean Ct of 19.04 ± 3.05. Whole blood samples showed slightly lower viral loads, with mean Ct of 21.63 ± 5.24. Serum and oral swab samples showed markedly lower viral loads, with mean Ct values of 25.37 ± 4.54 and 29.05 ± 2.41, respectively (Figure 5).

### 3.2. Performance of the Immunochromatography Assay

**Figure 6:**
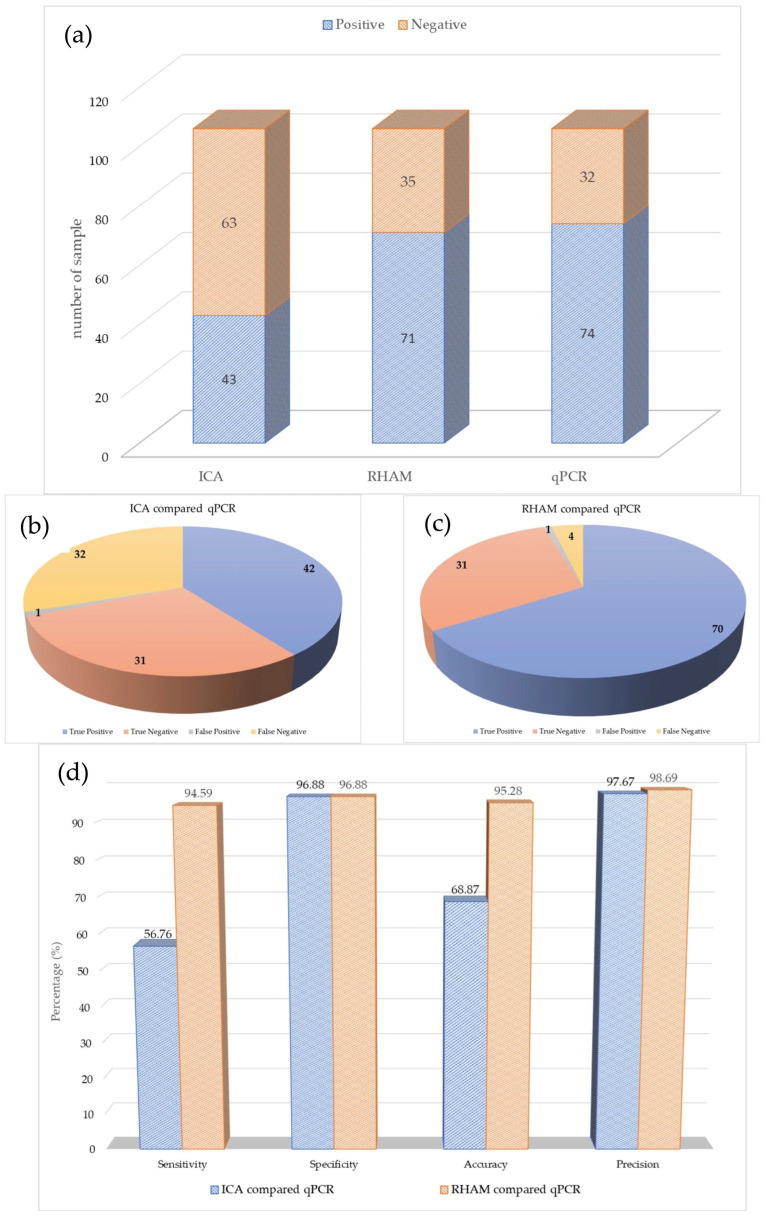
Performance graphs of ASFV detection using the immunochromatographic assay (ICA) and the RNase hybridization-assisted amplification (RHAM) ASFV test kit, compared with quantitative polymerase chain reaction (qPCR). (**a**) The ratio of positive and negative results obtained by each assay. (**b**,**c**) Comparison of the performance of the ICA and RHAM ASFV test kit with qPCR. (**d**) The analytical sensitivity, specificity, accuracy and precision of ICA and RHAM.

When compared with qPCR, the ICA correctly identified 42 of the 74 positive samples, resulting in 32 false-negative results. Additionally, the assays correctly identified 31 of 32 qPCR-negative samples, with one false positive (Figure 6). Further analysis of the false-negatives revealed that most corresponded to high qPCR Ct values (>27), suggesting an association between low viral load and detection failure (Table 1).

Based on these results, the ICA had a diagnostic sensitivity of 56.76% (95% CI: 45.48–68.04%) and a specificity of 96.88% (95% CI: 90.85–100%). The overall accuracy and precision of the ICA were 68.87% (95% CI: 60.06–77.68%) and 97.67% (95% CI: 93.18–100%), respectively, summarizing its test performance (Figure 6).

### 3.3. Performance of the RNase Hybridization-Assisted Amplification Test

Table 2 showed the results of ASFV genomic detection using the RHAM ASFV test kit in comparison with qPCR. The RHAM test not only provided positive or negative results but also reported detection duration, which correlated with the Ct values obtained by qPCR across all 106 samples. Negative results using RHAM were obtained within approximately 35 min. The 70 positive samples had a mean detection time of 15.85 min (range: 10.37–30.21 min).

The RHAM assay showed a strong overall concordance with the qPCR results. It correctly identified 70 of the 74 positive samples, with only four false-negative results. These false negatives occurred in samples with very high qPCR Ct values (33.51, 30.41, 33.77, and 30.38), indicating low viral loads (Sample 9, 10, 26 and 65). The assay produced one false-positive result (Sample 17) out of the 32 negative samples (Table 2).

Overall, this performance corresponds to a diagnostic sensitivity of 94.59% (95% CI: 89.42–99.76%) and a specificity of 96.88% (95% CI: 90.85–100%) for the RHAM assay. The overall accuracy and precision of the RHAM assay were 95.28% (95% CI: 91.24–99.32%), and 98.59% (95% CI: 95.85–100%), respectively (Figure 6).

### 3.4. Statistic Comparison of Assays

The level of agreement between each assay (ICA and RHAM) and the qPCR reference standard was assessed based on comparative statistical analysis. The RHAM assay demonstrated almost perfect agreement (κ = 0.891), whereas the ICA showed only moderate agreement (κ = 0.421).

McNemar’s test was used to directly compare the diagnostic performance of the two assays using paired samples. For diagnostic sensitivity, analysis of the 74 qPCR-positive samples indicated that RHAM’s superior performance was statistically significant (*p* < 0.0001). Specifically, RHAM correctly identified 28 positive samples that the ICA failed to detect. In contrast, analysis of the 32 qPCR-negative samples revealed no statistically significant difference in diagnostic specificity between the two assays (*p* = 1.0).

## 4. Discussion

In the absence of an effective ASFV vaccine, rapid and reliable diagnostics are essential to safeguard global pig populations [17]. It must be emphasized that the foundation of ASF surveillance and control, according to international guidelines from WOAH and FAO, relies on laboratory-based confirmation, with qPCR serving as the indispensable gold standard [9,10]. Field-deployable or penside assays, including those evaluated in this study, are not substitutes for laboratory diagnosis. Instead, they serve as critical screening tools intended strictly for rapid, preliminary investigation during active outbreaks, and their results (both positive and negative) must always be confirmed by laboratory methods. Differentiating ASF from other swine diseases is challenging because its clinical signs and post-mortem lesions often resemble those of classical swine fever (CSF) and highly pathogenic porcine reproductive and respiratory syndrome (HP-PRRS). These diseases frequently present with similar hemorrhagic lymph node lesions [18,19,20,21,22], which further complicates field diagnosis and underscores the need for highly accurate, specific molecular tools [23,24,25,26,27].

This study highlights a major limitation of the commonly used ICA, with a diagnostic sensitivity of only 56.76%. Detailed sample analysis indicates that ICA performance is strongly influenced by viral load (genome presence). The 32 false-negative samples were largely associated with high qPCR Ct values, most exceeding Ct 27. This suggests that the ICA fails to detect early infections, asymptomatic carriers, or samples with moderate to low viral antigen levels. These findings align with previous reports showing variable and often low sensitivity of ICA for ASFV antigen detection, particularly under field conditions where viral loads fluctuate [11]. Although ICA demonstrated 96.88% specificity, its poor sensitivity poses an epidemiological risk by potentially allowing undetected spread of the virus. Therefore, ICA use should be restricted to rapid confirmation of clinically symptomatic cases during outbreaks, where viral titers are typically high, rather than for definitive surveillance or control programs.

The RHAM assay demonstrated excellent diagnostic performance, with 94.6% sensitivity and 96.88% specificity, closely matching qPCR performance. The four false negatives occurred in samples with very high Ct values (>30), near the detection limit of most assays including qPCR, indicating that RHAM’s performance is nearly equivalent to the reference standard. Notably, a single false-positive result from the RHAM test (sample 17) was also detected as positive by the ICA, despite testing negative by qPCR. This discrepancy could stem from potential sample cross-contamination during collection or processing. Alternatively, both RHAM and ICA may have detected a true positive missed by the qPCR protocol used—a phenomenon occasionally reported when comparing different molecular assays [28]. Further investigation of such discordant samples is warranted to clarify these discrepancies. However, the original sample was exhausted after testing by ICA, RHAM and qPCR; therefore, it was unavailable for repeat qPCR or sequencing confirmation.

Despite these promising results, this study represents only a preliminary evaluation and thus has certain limitations. The validation was based on 106 clinical samples collected in Thailand, primarily from suspected cases, rather than including a comprehensive set of confirmed negative controls. Moreover, the genetic variability of ASFV, particularly the emergence of distinct genotypes (e.g., Genotype I and II), may influence the performance of molecular assays. This study did not assess the RHAM assay’s efficacy across different ASFV genotypes. Therefore, larger-scale validation studies using more diverse sample sets—including confirmed negative populations and multiple viral genotypes—are necessary to establish the assay’s diagnostic reliability in varied epidemiological settings.

Furthermore, this study underscores the critical importance of sample selection in ASFV diagnostics, which reflects the virus’s pathogenesis. The data in Table 1 show that the highest viral loads (lowest mean Ct values) were detected in pooled internal organs (lung, spleen, lymph node) (Ct 19.04), followed by whole blood (Ct 21.63). This aligns with the known pathophysiology of ASFV, which is characterized by an early viremia (high viral loads in blood) followed by extensive viral accumulation and replication in lymphoid tissues (such as the spleen and lymph nodes) and other secondary target organs [19]. Conversely, pooled oral swabs yielded the highest mean Ct value (29.05), indicating lower viral shedding through this route. However, this result must be interpreted with caution, as the samples were pooled (five swabs per sample), which may have diluted the viral concentration compared to an individual swab. This finding emphasizes that non-invasive sampling via oral swabs, while advantageous for screening, necessitates the use of highly sensitive molecular assays such as RHAM or qPCR to minimize the risk of false negatives.

The RHAM ASFV test kit employs isothermal amplification coupled with enzyme digestion probe technology to detect ASFV-specific sequences, providing a rapid and accurate diagnostic alternative. This assay integrates LAMP with an RNase HII reporter system for real-time signal visualization within a single reaction [29,30,31,32]. It targets the conserved B646L (p72) gene—also utilized in qPCR—which enhances analytical comparability and detection reliability [12,23,33]. Using conventional LAMP primers, Bst DNA polymerase exponentially amplifies the target sequence during isothermal incubation. A fluorescent ribonucleotide-containing probe, labeled with a 5′-fluorophore and 3′-quencher, hybridizes to the amplicon sequence. RNase HII then cleaves the ribonucleotide, releasing the probe and producing a detectable fluorescence signal. The Pluslife Integrated Nucleic Acid Testing Device records this fluorescence signal within 10–35 min, facilitating rapid ASFV detection in a single step. The assay incorporates an internal control to verify sample integrity and amplification efficiency, thereby minimizing false negatives and enhancing reliability. Collectively, this approach provides a versatile, efficient, and timely diagnostic platform that supports ASFV surveillance and rapid outbreak response.

Our results, based on diverse sample types including oral swab, whole blood, serum, tissue, and internal organs, demonstrated that the RHAM ASFV test kit achieved high sensitivity, specificity, accuracy, and precision when compared to qPCR. Despite this strong overall performance, four false-negative results were observed—two from oral swabs, one from serum, and one from whole blood—indicating potential limitations in assay sensitivity. These false-negative samples tested positive results in qPCR, with Ct value exceeding 30 (33.51, 30.41. 33.77, and 30.38), consistent with moderate to low viral genome levels approaching the detection limit of the RHAM assay. It is important to clarify that Ct values reflect the quantity of viral genome present, not necessarily the presence of infectious virus or active replications; typically, only Ct values above 35 are considered indicative of very ASFV genome levels. These findings suggest that the false negatives likely resulted from viral genome concentrations that fall below the assay’s detection threshold. Furthermore, the relatively small sample volume used in RHAM testing—approximately 150 μl collected via cotton swabbing—may have contributed to the occurrence of false negatives. In addition, qPCR involves genomic extraction prior to amplification, yielding a higher concentration of purified viral nucleic acid than RHAM and potentially explaining the discrepancy. Collectively, these factors, including differences in sample volume and nucleic acid concentration, likely account for the observed false negatives, as RHAM utilizes roughly half the sample volume used in qPCR, which may influence both sensitivity and specificity.

Currently, qPCR serves the primary molecular diagnostic method for detecting ASF [23,33], a disease with significant implications for the swine industry. Despite its sensitivity, qPCR’s dependence on specialized equipment and infrastructure limits accessibility and delays result turnaround. Consequently, samples must be transported to centralized laboratories, leading to delays and additional logistical challenges. Notably, qPCR requires nucleic acid extraction and amplification, taking at least 2.0 hr, whereas the RHAM assay provides results within 10–35 min, offering a significant advantage in speed and efficiency. In our study, negative results using RHAM were obtained within 35 min. The 70 positive samples showed a mean testing time of 15.85 min (range: 10.37–30.21 min), underscoring the assay’s rapidity and convenience compared with qPCR. Importantly, the time required for a positive RHAM result correlated with the qPCR Ct value, reflecting the relationship between testing time and viral load. Samples with lower Ct values—indicative of higher virus titers—produced shorter RHAM testing times, demonstrating its ability to detect high viral loads rapidly and underscoring its diagnostic efficacy. Overall, these findings indicate that RHAM functions as a semi-quantitative assay, enabling rapid and efficient ASFV detection while providing insight into viral load levels.

Several studies [34,35,36,37,38] have demonstrated that various isothermal methods, such as LAMP, recombinase polymerase amplification (RPA), and cross-priming amplification (CPA), enables sensitive and reliable detection of ASFV, underscoring its versatility in molecular diagnostics. Building upon this isothermal principle, the RHAM ASFV test kit functions as an on-site nucleic acid detection tool that combines LAMP-based amplification with a qPCR-like reporting system, enabling accurate and efficient ASFV detection. This integration of RHAM technology positions it within this growing landscape of field-deployable molecular tools, aiming to minimize false positives and enhances the overall reliability of test results. Additionally, the kit does not require refrigeration and provides results within 35 min, making it highly practical for rapid, field-based diagnostics. Collectively, these features establish the RHAM ASFV test kit as an accurate, rapid, and practical diagnostic platform, marking a meaningful advancement in molecular tools for animal disease surveillance and control.

Finally, the role of the RHAM assay should be considered within the broader context of a comprehensive field surveillance strategy. According to recommendations from the FAO and WOAH, effective disease control relies on the integration of multiple diagnostic approaches. On-site molecular assays, such as RHAM, are particularly effective for detecting viral genomes during the acute phase of infection. However, under field conditions—especially when assessing herd status or identifying survivors—molecular assays such as RHAM should be validated by qPCR and complemented by serological testing. This occurs because the sensitivity of viral genome detection methods (such as RHAM or qPCR) decreases after the acute phase, as animals recover and clear viremia, while antibodies remain detectable [39,40]. Therefore, combining molecular and serological diagnostics is essential for identifying both acute infections and survivors, thus providing a comprehensive epidemiological overview.

## 5. Conclusions

Effective African Swine Fever control is critically hampered by the diagnostic gap between slow, laboratory-bound qPCR and fast but unreliable field-based ICAs. This study evaluated the novel RHAM technology as a high-sensitivity, point-of-need solution. Based on 106 clinical field samples, RHAM demonstrated outstanding performance, achieving 94.59% diagnostic sensitivity and 96.88% specificity, showing “almost perfect agreement” (κ = 0.891) with the qPCR reference standard. This was statistically superior (*p* < 0.0001) to the conventional ICA, which showed poor sensitivity (56.76%) and failed to detect over 40% of positive cases. RHAM provides these results rapidly (10–35 min) by combining isothermal amplification with a specific RNase HII probe mechanism. Although this preliminary study requires further validation across different genotypes and geographical regions, we conclude that RHAM is a powerful, accurate, and field-deployable tool, representing a significant advancement for the rapid detection and timely containment of ASF outbreaks.

## Figures and Tables

**Table 1:** Comparative results of p72 antigen detection using immunochromatographic assay (ICA) and genomic detection using quantitative polymerase chain reaction (qPCR) for ASFV.

No.	ICA	qPCR		No.	ICA	qPCR		No.	ICA	qPCR	
	Result	Result	Ct		Result	Result	Ct		Result	Result	Ct
1	positive	positive	24.51	37	positive	positive	23.59	73	negative	negative	-
2	negative	positive	30.21	38	positive	positive	24.25	74	negative	negative	-
3	negative	positive	28.77	39	negative	positive	28.13	75	negative	negative	-
4	negative	positive	27.55	40	negative	positive	31.43	76	negative	negative	-
5	negative	positive	27.33	41	negative	positive	35.01	77	negative	negative	-
6	negative	positive	29.75	42	negative	negative	-	78	negative	negative	-
7	negative	positive	28.11	43	negative	negative	-	79	negative	negative	-
8	negative	positive	30.37	44	negative	negative	-	80	negative	negative	-
9	negative	positive	33.51	45	positive	positive	19.81	81	negative	negative	-
10	negative	positive	30.41	46	positive	positive	18.61	82	negative	negative	-
11	negative	negative	-	47	positive	positive	24.32	83	positive	positive	24.74
12	negative	negative	-	48	positive	positive	25.53	84	positive	positive	23.42
13	positive	positive	17.26	49	positive	positive	17.18	85	negative	positive	28.51
14	positive	positive	20.52	50	negative	negative	-	86	positive	positive	22.54
15	positive	positive	23.98	51	positive	positive	16.24	87	negative	positive	29.39
16	negative	positive	28.02	52	positive	positive	19.3	88	negative	positive	28.37
17	positive	negative	-	53	positive	positive	18.59	89	positive	positive	26.31
18	negative	negative	-	54	positive	positive	16.35	90	negative	positive	30.17
19	negative	negative	-	55	positive	positive	18.37	91	negative	positive	28.58
20	negative	negative	-	56	positive	positive	15.85	92	negative	positive	28.19
21	positive	positive	18.03	57	positive	positive	15.68	93	negative	positive	32.21
22	positive	positive	19.06	58	positive	positive	17.66	94	negative	negative	-
23	positive	positive	22.40	59	positive	positive	26.08	95	negative	negative	-
24	positive	positive	26.51	60	positive	positive	20.85	96	negative	negative	-
25	negative	positive	30.14	61	negative	negative	-	97	negative	negative	-
26	negative	positive	33.77	62	negative	negative	-	98	negative	negative	-s
27	negative	negative	-	63	positive	positive	25.79	99	positive	positive	21.08
28	negative	negative	-	64	positive	positive	24.61	100	positive	positive	19.49
29	positive	positive	17.29	65	negative	positive	30.38	101	positive	positive	17.58
30	positive	positive	18.34	66	positive	positive	22.85	102	positive	positive	31.98
31	positive	positive	21.04	67	negative	positive	30.59	103	positive	positive	17.91
32	positive	positive	24.28	68	negative	positive	29.35	104	positive	positive	22.68
33	negative	positive	28.27	69	negative	positive	27.22	105	negative	positive	27.02
34	negative	positive	31.86	70	negative	positive	30.09	106	positive	positive	21.83
35	negative	negative	-	71	negative	positive	29.50				
36	negative	negative	-	72	negative	negative	-				

Ct: cycle threshold; positive: detected antigen or nucleic acid; negative: undetected antigen or nucleic acid.

**Table 2:** Results of genomic detection of ASFV using the RNase hybridization-assisted amplification (RHAM) ASFV test kit compared with quantitative polymerase chain reaction (qPCR).

No.	RHAM		qPCR		No.	RHAM		qPCR		No.	RHAM		qPCR	
	Result	Min	Result	Ct		Result	Min	Result	Ct		Result	Min	Result	Ct
1	positive	13.37	positive	24.51	37	positive	13.34	positive	23.59	73	negative	35.00	negative	-
2	positive	13.35	positive	30.21	38	positive	13.34	positive	24.25	74	negative	35.00	negative	-
3	positive	13.42	positive	28.77	39	positive	14.34	positive	28.13	75	negative	35.00	negative	-
4	positive	13.35	positive	27.55	40	positive	16.33	positive	31.43	76	negative	35.00	negative	-
5	positive	13.37	positive	27.33	41	positive	22.29	positive	35.01	77	negative	35.00	negative	-
6	positive	14.34	positive	29.75	42	negative	35.00	negative	-	78	negative	35.00	negative	-
7	positive	13.35	positive	28.11	43	negative	35.00	negative	-	79	negative	35.00	negative	-
8	positive	20.40	positive	30.37	44	negative	35.00	negative	-	80	negative	35.00	negative	-
9	negative	35.00	positive	33.51	45	positive	10.37	positive	19.81	81	negative	35.00	negative	-
10	negative	35.00	positive	30.41	46	positive	11.36	positive	18.61	82	negative	35.00	negative	-
11	negative	35.00	negative	-	47	positive	13.35	positive	24.32	83	positive	26.37	positive	24.74
12	negative	35.00	negative	-	48	positive	12.36	positive	25.53	84	positive	17.31	positive	23.42
13	positive	11.45	positive	17.26	49	positive	16.31	positive	17.18	85	positive	15.33	positive	28.51
14	positive	19.30	positive	20.52	50	negative	35.00	negative	-	86	positive	22.32	positive	22.54
15	positive	15.33	positive	23.98	51	positive	12.35	positive	16.24	87	positive	17.32	positive	29.39
16	positive	15.33	positive	28.02	52	positive	20.33	positive	19.3	88	positive	18.31	positive	28.37
17	positive	18.33	negative	-	53	positive	14.35	positive	18.59	89	positive	15.33	positive	26.31
18	negative	35.00	negative	-	54	positive	11.42	positive	16.35	90	positive	20.28	positive	30.17
19	negative	35.00	negative	-	55	positive	15.33	positive	18.37	91	positive	15.33	positive	28.58
20	negative	35.00	negative	-	56	positive	12.38	positive	15.85	92	positive	15.33	positive	28.19
21	positive	15.14	positive	18.03	57	positive	12.36	positive	15.68	93	positive	18.30	positive	32.21
22	positive	11.37	positive	19.06	58	positive	16.41	positive	17.66	94	negative	35.00	negative	-
23	positive	11.39	positive	22.4	59	positive	24.25	positive	26.08	95	negative	35.00	negative	-
24	positive	12.36	positive	26.51	60	positive	20.33	positive	20.85	96	negative	35.00	negative	-
25	positive	12.36	positive	30.14	61	negative	35.00	negative	-	97	negative	35.00	negative	-
26	negative	35.00	positive	33.77	62	negative	35.00	negative	-	98	negative	35.00	negative	-
27	negative	35.00	negative	-	63	positive	16.32	positive	25.79	99	positive	14.34	positive	21.08
28	negative	35.00	negative	-	64	positive	19.34	positive	24.61	100	positive	12.36	positive	19.49
29	positive	14.35	positive	17.29	65	negative	35.00	positive	30.38	101	positive	11.37	positive	17.58
30	positive	18.31	positive	18.34	66	positive	17.36	positive	22.85	102	positive	15.33	positive	31.98
31	positive	13.36	positive	21.04	67	positive	20.29	positive	30.59	103	positive	13.35	positive	17.91
32	positive	13.36	positive	24.28	68	positive	30.21	positive	29.35	104	positive	13.35	positive	22.68
33	positive	15.34	positive	28.27	69	positive	17.40	positive	27.22	105	positive	16.33	positive	27.02
34	positive	21.29	positive	31.86	70	positive	20.32	positive	30.09	106	positive	13.35	positive	21.83
35	negative	35.00	negative	-	71	positive	19.30	positive	29.50					
36	negative	35.00	negative	-	72	negative	35.00	negative	-					

Ct: cycle threshold; positive: detected nucleic acid; negative: undetected nucleic acid.

## Data Availability

The original contributions presented in this study are included in the article material. Further inquiries can be directed to the corresponding author.

## References

[1] Costard S., Wieland B., De Glanville W., Jori F., Rowlands R., Vosloo W., Roger F., Pfeiffer D.U., Dixon L.K. (2009). African Swine fever: How can global spread be prevented?. Philos. Trans. R. Soc. B Biol. Sci..

[2] Sauter-Louis C., Conraths F.J., Probst C., Blohm U., Schulz K., Sehl J., Fischer M., Forth J.H., Zani L., Depner K. (2021). African Swine fever in wild boar in Europe—A review. Viruses.

[3] Ramirez-Medina E., O’Donnell V., Silva E., Esoinoza N., Velazquez-Salinas L., Moran K., Daite D.A., Barrette R., FAburay B., Holland R. (2022). Experimental infection of domestic pigs with an African swine fever virus field strain isolated in 2021 from the Dominican Republic. Viruses.

[4] Taesuji M., Rattanamas K., Kulthonggate U., Mamom T., Ruenphet S. (2022). Sensitivity and specificity for African horse sickness antibodies detection using monovalent and polyvalent vaccine antigen-based dot blotting. Vet. World.

[5] Rattanamas K., Taesuji M., Kulthonggate U., Jantafong T., Mamom T., Ruenphet S. (2022). Sensitivity of RNA viral nucleic acid-based detection of avian influenza virus, Newcastle disease virus, and African horse sickness virus on flinders technology associates card using conventional reverse-transcription polymerase chain reaction. Vet. World.

[6] Nguyen-Thi T., Pham-Thi-Ngoc L., Nguyen-Ngoc Q., Dang-Xuan S., Lee H.S., Nguyen-Viet H., Padungtod P., Nguyen-Thu T., Nguyen-Thi T., Tran-Cong T. (2021). An assessment of the economic impacts of the 2019 African swine fever outbreaks in Vietnam. Front. Vet. Sci..

[7] Ata E.B., Li Z.J., Shi C.W., Yang G.L., Yang W.T., Wang C.F. (2022). African Swine Fever Virus: A Raised Global Upsurge and a Continuous Threaten to Pig Husbandry. Microb. Pathog..

[8] World Organisation for Animal Health (WOAH) (2016). Chapter 15.1. Infection with African Swine Fever Virus. Terrestrial Animal Health Code.

[9] Beltrán-Alcrudo D., Arias M., Gallardo C., Kramer S., Penrith M.L. (2017). African Swine Fever: Detection and Diagnosis—A Manual for Veterinarians. FAO Animal Production and Health Manual No. 19.

[10] Dixon L.K., Stahl K., Jori F., Vial L., Pfeiffer D.U. (2020). African Swine Fever Epidemiology and Control. Annu. Rev. Anim. Biosci..

[11] Fernández-Pinero J., Gallardo C., Elizalde M., Robles A., Gomez C., Bishop R., Heath L., Couacy-Hymann E., Fasina F.O., Pelayo V. (2013). Molecular diagnosis of African swine fever by a new real-time PCR using universal probe library. Transbound. Emerg. Dis..

[12] Gallardo C., Soler A., Nurmoja I., Cano-Gomez C., Cvetkova S., Frant M., Wozniakowski G., Simon A., Perez C., Nieto R. (2021). Dynamics of African swine fever virus (ASFV) infection in domestic pigs infected with virulent, moderate virulent and attenuated genotype II ASFV European isolates. Transbound. Emerg. Dis..

[13] Wang S., Shen H., Lin Q., Huang J., Zhang C., Liu Z., Sun M., Zhang J., Liao M., Li Y. (2022). Development of a. cleaved probe-based loop-mediated isothermal amplification assay for rapid detection of African swine fever virus. Front. Cell. Infect. Microbiol..

[14] Prasitsuwan W., Suwannachote T., Sumalai T., Lertpatarakomol R., Trairatapiwan T., Ruenphart S. (2025). A comparison of diagnostic methods for canine Ehrlichiosis: Microscopy and RNases hybridization-assisted amplification technology compared with the quantitative polymerase chain reaction. Vet. World.

[15] Punyadarsaniya D., Taesuji M., Rattanamas K., Ruenphet S. (2025). Establishment of an In-House Indirect Enzyme-Linked Immunosorbent Assay to Detect Antibodies Against African Horse Sickness Based on Monovalent and Polyvalent Live Attenuated Vaccines During the First Outbreak in Thailand. Animals.

[16] Suwannachote T., Prasitsuwan W., Sumalai T., Ruenphet S. (2025). A Comparison of Diagnostic Methods for Feline Leukemia Virus and Feline Immunodeficiency Virus: Immunochromatographic Assay and RNases Hybridization-Assisted Amplification Test Kit Compared to Reverse Transcription Quantitative Polymerase Chain Reaction. Animals.

[17] Tran X.H., Phuong L.T., Huy N.Q., Thuy D.T., Nguyen V.D., Quang P.H., Ngon Q.V., Rai A., Gay C.G., Gladue D.P. (2022). Evaluation of the Safety Profile of the ASFV Vaccine Candidate ASFV-G-∆I177L. Viruses.

[18] Gallardo C., Soler A., Nieto R., Cano C., Pelayo V., Sanchez M.A., Pridotkas G., Fernandez-Pinero J., Briones V., Arias M. (2017). Experimental infection of domestic pigs with African swine fever virus Lithuania 2014 genotype II field isolate. Transbound. Emerg. Dis..

[19] Salguero F.J., Frossard J.P., Rebel J.M., Stadejek T., Morgan S.B., Graham S.P., Steinbach F. (2015). Host-pathogen interactions during porcine reproductive and respiratory syndrome virus 1 infection of piglets. Virus Res..

[20] Morgan S.B., Frossard J.P., Pallares F.J., Gough J., Stadejek T., Graham S.P., Steinbach F., Drew T.W., Salguero F.J. (2016). Pathology and virus distribution in the lung and lymphoid tissues of pigs experimentally inoculated with three distinct type 1 PRRS virus isolates of varying pathogenicity. Transbound. Emerg. Dis..

[21] Huong Giang N.T., Lan N.T., Nam N.H., Hirai T., Yamaguchi R. (2016). Pathological characterization of an outbreak of porcine reproductive and respiratory syndrome in Northern Vietnam. J. Comp. Pathol..

[22] Choe S., Le V.P., Shin J., Kim J.H., Kim K.S., Song S., Cha R.M., Park G.N., Nguyen T.L., Hyun B.H. (2020). Pathogenicity and genetic characterization of Vietnamese classical swine fever virus: 2014–2018. Pathogens.

[23] King D.P., Reid S.M., Hutchings G.H., Grierson S.S., Wilkinson P.J., Dixon L.K., Bastos A.D.S., Drew T.W. (2003). Development of a TaqMan^®^ PCR assay with internal amplification control for the detection of African swine fever virus. J. Virol. Methods.

[24] Notomi T., Okayama H., Masubuchi H., Yonekawa T., Watanabe K., Amino N., Hase T. (2000). Loop-mediated isothermal amplification of DNA. Nucleic Acids Res..

[25] Rowlands R.J., Duarte M.M., Boinas F., Hutchings G., Dixon L.K. (2009). The CD2v protein enhances African swine fever virus replication in the tick vector, *Ornithodoros erraticus*. Virology.

[26] Taesuji M., Rattanamas K., Punyadarsaniya D., Mamom T., Nguyen H.T., Ruenphet S. (2021). In vitro primary porcine alveolar macrophage cell toxicity and African swine fever virus inactivation using five commercially supply compound disinfectants under various condition. J. Vet. Med. Sci..

[27] Sovijit W., Taesuji W., Rattanamas K., Punyadarsaniya D., Mamom T., Nguyen H.T., Ruenphet S. (2021). In vitro cytotoxicity and virucidal efficacy against African swine fever using two potassium hydrogen peroxymonosulfate compared to a quaternary ammonium compound under various concentrations, exposure times and temperatures. Vet. World.

[28] Petrovan V., Buburuzan L., Zaulet M. (2015). False positive results using PCR detection method for African swine fever virus in wild boars from northern Romanian hunting zones. Turk. J. Vet. Anim. Sci..

[29] Zhu D., Huang J., Hu B., Cao D., Chen D., Song X., Chen J., Zhou H., Cen A., Hou T. (2023). Trial of the Pluslife SARS-CoV-2 Nucleic Acid Rapid Test Kit: Prospective Cohort Study. JMIR Public Health Surveill..

[30] Xiao Z., Liu X., Kang X., Feng Y., Zheng L., Chen C. (2023). Rapid and accurate detection of SARS-CoV-2 using the RHAM technology. Sci. Rep..

[31] Herrmann L., Breuer J., Duc T.N., Thomé N., Ghazaani F., Kamhieh-Milz S., Pfützner A. (2024). Comparison of the diagnostic accuracy of the Pluslife Mini Dock RHAM technology with Abbott ID Now and Cepheid GenXpert: A retrospective evaluation study. Sci. Rep..

[32] Zhong G., Zhao Z., He Y., Yu X., Yang T., Liu H., Huang B. (2025). Evaluation of the RHAM-based Pluslife Rapid SARS-CoV-2 assay and comparison with EasyNAT Rapid SARS-CoV-2 assay and Wondfo 2019-nCoV antigen assay. Microbiol. Spectr..

[33] Zsak L., Borca M.V., Risatti G.R., Zsak A., French R.A., Kutish G.F., Neilan J.G., Callahan J.D., Nelson W.M., Rock D.L. (2005). Preclinical diagnosis of African swine fever in contact-exposed swine by a real-time PCR assay. J. Clin. Microbiol..

[34] Bohorquez J.A., Lanka S., Rosell R., Perez-Simo M., Alberch M., Rodriguez F., Ganges L., Maddox C.W. (2023). Efficient detection of African Swine Fever Virus using minimal equipment through a LAMP PCR method. Front. Cell. Infect. Microbiol..

[35] Mee P.T., Wong S., O’Riley K.J., da Conceicao F., da Costa Jong J.B., Phillips D.E., Rodoni B.C., Rawlin G.T., Lynch S.E. (2020). Field Verification of an African Swine Fever Virus Loop-Mediated Isothermal Amplification (LAMP) Assay during an Outbreak in Timor-Leste. Viruses.

[36] Wang Y., Dai J.F., Liu Y.S., Yang J., Hou Q., Ou Y., Ding Y., Ma B., Chen H., Li M.M. (2021). Development of a Potential Penside Colorimetric LAMP Assay Using Neutral Red for Detection of African Swine Fever Virus. Front. Microbiol..

[37] Zuo L., Song Z., Zhang Y., Zhai X., Zhai Y., Mei X., Yang X., Wang H. (2020). Loop-Mediated Isothemal Amplification Combined with Lateral Flow Dipstrick for On-Site Diagnosis of African Swine Fever Virus. Virol. Sin..

[38] Ji C., Zhou L., Chen Y., Fang X., Liu Y., Du M., Lu X., Li Q., Wang H., Sun Y. (2023). Microfluidic-LAMP chip for the point-of-care detection of gene-deleted and wild-type African swine fever viruses and other four swine pathogens. Front. Vet. Sci..

[39] Gallardo C., Nieto R., Soler A., Pelayo V., Fernández-Pinero J., Markowska-Daniel I., Arias M. (2015). Assessment of African swine fever diagnostic techniques as a response to the epidemic outbreaks in eastern european union countries: How to improve surveillance and control programs. J. Clin. Microbiol..

[40] Oura C.A.L., Edwards L., Batten C.A. (2013). Virological diagnosis of African swine fever—Comparative study of available tests. Virus Res..

